# Minimally Invasive Surgery for the Treatment of Lumbar Spinal Stenosis: A Comprehensive Review

**DOI:** 10.1007/s11916-026-01554-9

**Published:** 2026-09-23

**Authors:** Francesco Sammartino, Tian Nancy, Hannah Ong, Breanna Santoso, Jayesh Vallabh, Kristen Noon

**Affiliations:** 1 Department of Anesthesiology, The Ohio State University, N411 Doan Hall, 410 W. 10th Ave, Columbus, OH 43210 USA; 2 Department of PMR, The Ohio State University, 410 Medical Center Dr, Columbus, OH USA; 3 School of Medicine, The Ohio State University, 1645 Neil Ave, Columbus, OH 43210 USA; 4 Heritage College of Osteopathic Medicine, Ohio University, 191 W Union St, OH 45701 Athens, USA

**Keywords:** Lumbar stenosis, Decompression, Neurogenic claudication, Outpatient laminectomy, Percutaneous image-guided lumbar decompression

## Abstract

**Introduction:**

Lumbar spinal stenosis (LSS) is a prevalent and debilitating condition characterized by the narrowing of the spinal canal, leading to pain and neurogenic claudication. While open laminectomy is the gold standard for severe cases, it carries significant risks, especially for older patients. The variable success of non-surgical conservative treatments highlights the need for l minimally invasive interventions. This review compares available surgical options for LSS, with a focus on percutaneous image-Guided lumbar decompression (PILD) and minimally invasive spine (MIS) procedures.

**Methods:**

A literature review of clinical studies on MIS procedures for lumbar stenosis published between 2000 and 2025 was conducted via PubMed.

**Results:**

The PILD procedure, which involves the percutaneous removal of hypertrophied ligamentum flavum, has demonstrated statistically significant reductions in pain and improvements in functional mobility in multiple studies, including three Level 1 randomized controlled trials. Pain relief emerges as early as six weeks and is sustained for up to two years, with a low rate of minor complications. Other MIS options include interspinous spacers and endoscopic decompression, which also show positive outcomes in select patient populations.

**Conclusion:**

The PILD procedure is a safe and effective MIS treatment for LSS specifically caused by ligamentum flavum hypertrophy in patients who have failed conservative care. While offering reduced morbidity compared to open surgery, its long-term efficacy relative to other MIS and traditional techniques requires further investigation.

## Introduction

Lumbar spinal stenosis (LSS) is defined as any narrowing in the spinal canal [1]. Anatomically, structures that can be affected include the central spinal canal, lateral recesses, or intervertebral foramen [1]. Though imaging findings of lumbar spinal stenosis can be asymptomatic, clinical manifestations typically present as pain, cramping, and radicular symptoms due to compression of neurovascular structures [1, 2]. As symptoms progress, patients may develop neurogenic claudication, further limiting their ability to ambulate and worsening patient function [1, 2]. A systematic review by Jensen et al. demonstrated that the prevalence of clinically diagnosed lumbar spinal stenosis is between 11 and 39% [3].

Lumbar spinal stenosis can be divided into acquired and congenital. Those with congenital etiology can have pre-existing narrowed spinal canals, and any mild degeneration can lead to worsening symptoms [4]. However, the vast majority of cases are acquired cases of stenosis. This includes degenerative changes. Imaging typically demonstrates disc degeneration, facet degeneration, ligamentum flavum hypertrophy, scoliosis, and ligament ossification [3]. Acquired etiologies also include post-surgery changes, traumatic, spondylolytic, metabolic changes, infectious, and rheumatologic etiologies [3].

Prognosis and symptom severity often vary widely, even among patients with similar MRI findings [5]. MRI grading systems such as the Schizas classification assess dural sac morphology from Grade A (no/minor stenosis) to Grade D (extreme stenosis) [6]. Patients with Grade C - D disease are more likely to worsen without surgical intervention, while many in Grades A - B remain stable or improve with conservative care [7, 8]. While rapid neurological decline is uncommon, the decision to pursue surgical intervention is often guided by symptom progression and functional status.

Long-term studies on conservative treatment show 33% of patients’ symptoms improve, 50% remain stable, and 10–20% worsen over 3 to 10 years [7–9]. Additionally, in a 3-year prospective study focused on moderate LSS, approximately 22% reported worsening walking ability and 10–13% reported increased pain without surgical intervention [9]. These studies highlight significant variability in the clinical course of LSS despite current prognostic measures.

Open laminectomy remains the gold-standard decompression intervention for severe LSS, with average Oswestry Disability Index (ODI) improvements of ~ 20% [1, 10]. However, these procedures carry substantial risks, particularly in older patients, including dural tears (5–9%), wound infection (1–4%), cardiopulmonary complications (2–3%), and readmission within 30 days (~ 7–10%) [10, 11]. The wide variability between radiologic severity and clinical prognosis, and the significant risks of open surgery, emphasize the need for interventions that can be offered even to patients in lower severity grades.

**Fig. 1:**
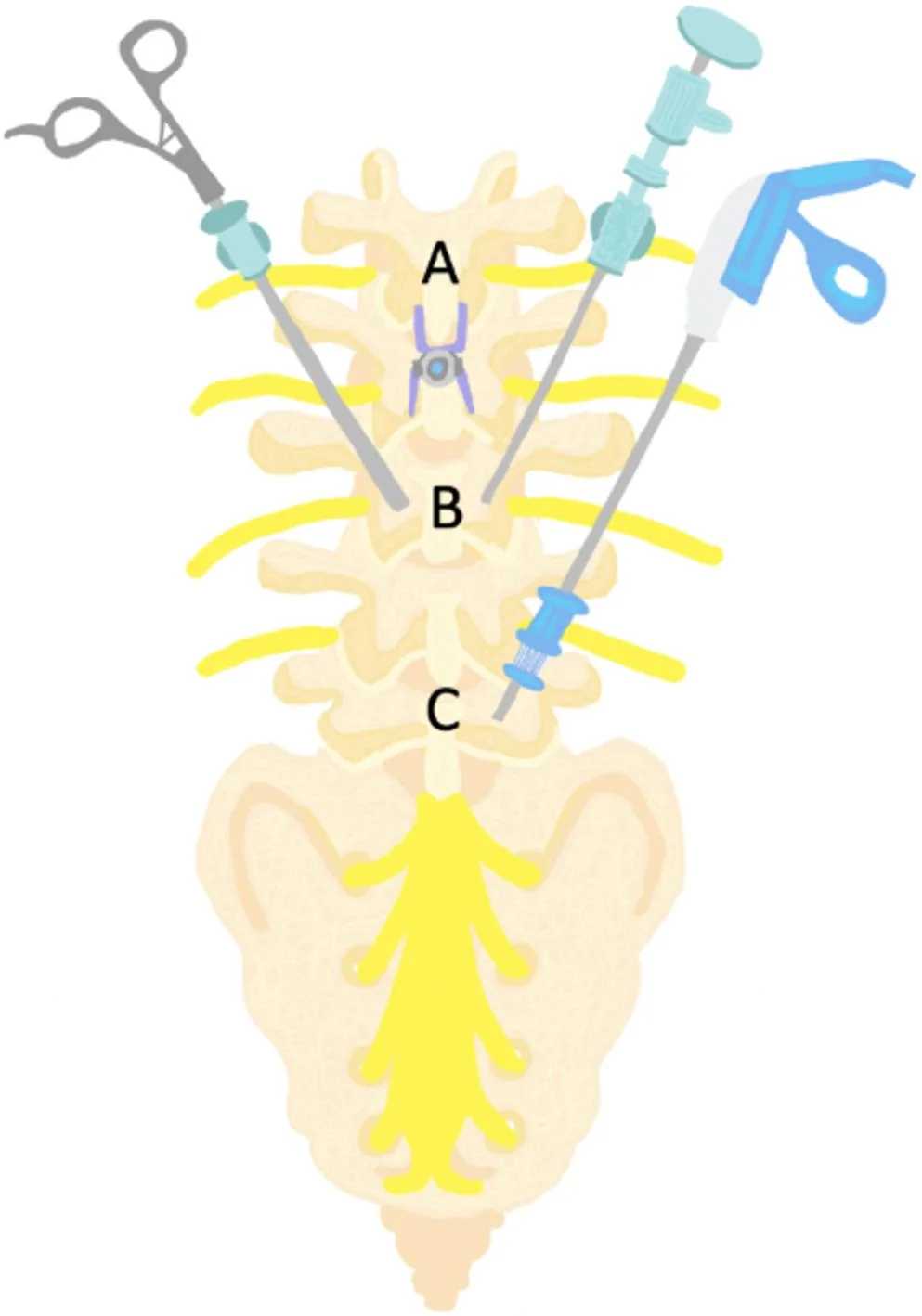
Pictorial representation of the 3 most common minimally-invasive surgical options for the treatment of lumbar stenosis, mainly interspinous devices (**A**), endoscopic spine surgery (**B**) and the mild^®^ procedure (**C**)

This review aims to compare the available surgical options for LSS, focusing on the MIS procedures, including the PILD procedure (Fig. 1).

## Current Evidence for Conservative Management in LSS

Typically, conservative treatment for the symptomatic management of LSS includes physical therapy, exercise, and stretching. Other nonsurgical treatments have included bracing, analgesic medications, lifestyle interventions, interlaminar (ESI) and transforaminal (TFESI) epidural steroid injections, and, in selected cases, lumbar medial branch radiofrequency ablation [12].

Despite the frequent use of epidural steroid injections (ESIs) for lumbar spinal stenosis (LSS), with 23% of all such injections administered for this condition, their effectiveness in the long term is supported by limited evidence [13]. 

The progressive nature of LSS, often involving a thickened ligamentum flavum that is mechanically unaffected by steroid injections, offers a potential explanation for the lack of consistent positive outcomes. For example, a systematic review by Chou et al. [14] concluded that ESIs provided no clear impact on symptoms related to spinal stenosis. Furthermore, a comprehensive 2020 review by the Cochrane Collaboration [1] found only limited support for the use of ESIs in patients with lumbosacral radicular pain, as the treatment effects are small, and they are mainly evident at short-term follow-up.

Overall, the efficacy of ESI in LSS is reported in most trials [15], although the wide variation in epidural technique, medication selection, dosage, and the duration of improvement makes it difficult to establish the long-term effectiveness of this modality.

While a rapid decline in neurological function is uncommon in individuals with lumbar spinal stenosis, delaying surgery in the presence of cauda equina/conus syndrome or other acute worsening neurological function can reduce the chances of symptom improvement. In general, the decision to undergo surgery is often elective and based on patient preference after weighing potential risks and benefits.

### The Percutaneous Image-Guided Lumbar Decompression Procedure

The percutaneous image-guided lumbar decompression procedure (PILD) has been successfully used in clinical practice for over a decade and involves obtaining posterolateral access to the interlaminar space through a small trocar portal. The first set of instruments was invented in 2005 by Dr. David Solsberg and Dr. Donald Schomer. It was first designed to treat LSS patients with malignant comorbidities who were unable to tolerate open surgery. It was named X-Sten mild→ Tool Kit and received FDA approval in December 2006. Until 2017, the lack of CMS and limited private-payer coverage rendered this procedure financially impractical, creating regional gaps; from 2017, CMS Coverage with Evidence Development (CED) reimbursed procedures within approved claims-analysis studies, accelerating adoption.

Patients are eligible for the PILD procedure if they have experienced lumbar spinal stenosis symptoms for more than three months—such as lumbar, buttock, or leg pain that worsens with standing or walking and improves when bending forward or sitting—provided they do not have contraindications like prior surgery at the treatment level, significant (Grade > 1) spondylolisthesis, symptomatic disc protrusion, excessive facet hypertrophy, bleeding disorders, or inability to maintain a prone position.

Currently, the most commonly used system is the Stryker (Stryker, Inc.) mild^®^, which uses a specialized surgical kit to remove portions of hypertrophied ligamentum flavum and lamina through a 5 mm skin incision, under local anesthesia [16, 17]. Using a combination of the contralateral oblique view (CLO) and anterior/posterior/lateral view for verification of positioning under the lamina, an epidurogram is used to identify the anterior margin of the ligamentum flavum. Trajectory is determined by using a 5″ 22G needle. A 6G mild^®^ portal cannula with trocar is used to access the soft tissue of the posterior lumbar spine. A bone sculpting rongeur is then used to remove portions of the lamina.

Following the epidurogram, the guiding portal and inner trocar can be advanced at the inferior lumbar segment and lateral to the spinous process margin to reach the ligamentum flavum. At this point, the inner trocar can be removed, and the portal can be secured to the skin using the back plate. The bone sculpting component can then be advanced if there is need to further enlarge the lamina edges, and prepare the field for the tissue sculpting component, which allows for ligament decompression. After this step, a repeat epidurogram can be used to confirm the extent of the decompression. The portal site is then closed with a sterile adhesive strip or glue.

A detailed comparison of the main clinical studies on mild^®^ is outlined in Table 1.

**Table 1:** Detailed analysis of the included trials

Author, year	Study design	Follow up	Number of patients	Outcomes assessment	Complications	Surgical time
Chopko, [18] MIDAS I study	Prospective cohort, multi-center	6 wks	75	At 6 weeks, the mild^®^ procedure showed statistically and clinically significant reduction in pain and improvement in function and mobility as measured by VAS [improvement of 3.6 points from baseline to 6-week], ODI [improvement of 17.9 points from baseline to 6 weeks], ZCQ, and SF-12v2	None	-
Chopko, [19]	Prospective, single site	4–72 wks	14	VAS improved [3.5 points or 52.6% from baseline] , no statistically significant improvement in ODI.	DVT/PE in 1 patient, small bowel herniation in 1 patient	-
Basu, [20]	Prospective, single site	6 months	27	VAS improved [improvement of 5.2 points from baseline] and ODI [24 points] , ZCQ also improved	None	1 h
Deer, [21]	Prospective, single site	12 wks, 6 months , 1 year	46	VAS improved [2.7 points at 12 wks , up to 2.9 points at 1 year], ODI improved [14.3 points at 12 months and up to 17.4 at 1 year], ZCQ improved	None	Mean treatment time was 41 min from patient entry to departure from the operating room. Fluoroscopy time ranged from 38 to 279 s, with a mean of 104 s and median of 98 s
Mekhail, [22]	Prospective, single center	1 year	40	VAS improved [improvement of 2.9 points], ODI improved [improvement of 11.9 points], ZCQ and SF-12v2 improved as well	None	1 h median
Mekhail, [23]	Prospective, multi-center	3 months, 6 months, 9 months, 1 year	40	VAS improved [reduction to almost half at 12 mo, biggest reduction at 3 mo] and PDI [reduction of 22.6 points at 12mo]	None	Mean fluoro time per patient ranged from 170 to 235 s
Wilkinson, [24]	Prospective, single center	1-6-12-26 wks	10	VAS decreased by 3 + points	average surgery time was 101.6 min	Recurrent claudication requiring laminectomy (*n* = 6), postoperative headache (*n* = 1), Transient worsening of left hip pain (*n* = 1)
Staats, [25]	Prospective randomized trial, multi-center	6 months	149 had mild, the rest 153 had ESI	NPRS decreased by > 2 points in 22% more patients than controls, ODI improved by 10 points in 18% more patients than controls	mean of 43.0 min for mild	None
Staats, [26]	Prospective randomized trial, multi-center	6 months, 1 year, 2 years	149	At 2 years, Oswestry Disability Index improved by 22.7 points, Numeric Pain Rating Scale improved by 3.6 points, and Zurich Claudication Questionnaire symptom severity and physical function domains improved by 1.0 and 0.8 points	same	None
Benyamin, [27]	Prospective randomized controlled trial	149 mild / 153 ESI	6 months and 1 year	58.0% ODI responder rate in the mild^®^ group was higher than the 27.1% responder rate in the epidural steroid group , same safety	same	None
Brown, 2012	Double blind, randomized, prospective, single site	38 (21 Mild, 17 ESI)	6 and 12 wks	VAS improved 2.5 points in the Mild group, and continued at 1 year	-	None
Pryzbylkowski, [28]	Retrospective single-center cohort study	145 (75 receiving ≤ 1 ESIs and 70 patients receiving ≥ 2 ESIs)	3 months	VAS improved 3 points in both groups		None
Pope [29]	Retrospective single-center cohort study	147 (80 receiving epidurogram and 67 not)	3 months	No complications	-	None
Lingreen, [30]	Retrospective Descriptive Study	44	1 month	Average VAS decreased 4 points		Minor adverse events like post-procedure soreness
Deer, 2021/2022/2024 “The MOTION study” [29, 31, 32]	Prospective, multicenter, randomized controlled trial	155 subjects randomized to CMM or mild^®^ +CMM	6 , 12 , 24 months	At 2 year follow-up, statistically significant improvement in ODI [14.6 points improvement] and NPRS [2 point for the low back and 4 point for the leg pain] were observed . Substantial crossover to CMM+mild^®^ hindered between-group comparisons	1 h	None
Chu, 2025	Retrospective, multisite	175 patients underwent mild^®^, 175 had open decompression, propensity score matched	Up to 24 months	Only 22.2% of the mild^®^ cohort achieved the MCID for pain improvement compared with 43.1% of the open decompression cohort Higher re-operation rate in the mild^®^ cohort (46.2% compared with 29.3%)	Mean operative time was longer in the open decompression group (190 ± 87 vs. 55 ± 24 min	mild^®^ cohort had higher rates of postoperative neurological deficits (6.3% vs. 0.6%, *p* = 0.003) (neuritis)

There are 3 level 1 RCT trials on mild^®^[21, 26, 32]. The most consistently reported outcomes were the change in baseline VAS and ODI scores. The reported patient mean (SD) age was 71.5 (10.8) years. The average surgical time was around 1 h for 1 level treated. Of note, the studies have different patient inclusion criteria, which may affect the generalizability of their results. In the MIDAS Encore study, for example, the authors included patients with spondylolisthesis Grade 2, while in the other studies, only patients with Grade 1 were included. Another discrepancy is related to the measured thickness of the ligamentum flavum, which was >4 mm in the original Mekhail’s study, but it was otherwise > 2.5 mm in the other studies. The MiDAS Encore study clearly outlines that only central stenosis was eligible for mild^®^; however, this criterion was not mentioned in any other article.

Few articles have described the quantity of debulked ligamentum, and several studies did not routinely perform epidurogram to quantify the extent of decompression [29].

Between 86.7% and 100% of participants were treated bilaterally, with about 50% of patients having 2 levels treated within the same session.

Regarding the trend of pain relief after mild^®^, as measured by VAS, this can emerge as early as 6 weeks postoperatively and remain stable for up to 5 years postoperatively in some series [33].

Lastly, mild^®^ reported complications were rare and minor, with only one study reporting dural tear and epidural hematoma [26].

### The Rise of MIS Surgery

Over the last decade, minimally invasive spine (MIS) surgery has become increasingly popular due to reduced soft tissue trauma and postoperative pain compared to open surgery. Studies have also indicated that MIS lumbar laminectomy may lead to better clinical outcomes and faster recovery than open lumbar decompression [34].

Traditionally, especially in urgent cases presenting with cauda equina/conus syndrome, which are overall rare, open laminectomy is the preferred approach.

In a large prospective trial with 12 months follow-up [35] enrolling elderly patients with LSS undergoing decompressive laminectomy, the mean VAS change for back and leg pain decreased by 18%, while the ODI improved by almost 50%. With the limitations related to using VAS as an outcome measure, there is a comparable similarity with the outcomes reported after mild^®^.

Currently, there is a trend in limiting surgical fusion to those cases of LSS where there is a need to correct deformity, stabilize painful segment movement, and restore lordosis and sagittal balance.

The Norwegian Degenerative Spondylolisthesis and Spinal Stenosis study (NORDSTEN study) [36] randomized 130 patients to laminectomy or laminectomy and fusion, and followed them up to 5 years. The authors found that the five-year results of decompression alone were non-inferior to those of decompression with instrumented fusion. A secondary analysis of patients with lumbar spinal stenosis with spondylolisthesis included in the NORDSTEN trial found that the common indications for performing fusion with decompression (i.e., instability) did not influence the primary outcome of ODI. Although the NORDSTEN trials weren’t designed to compare outcomes in spinal stenosis patients with and without spondylolisthesis, findings suggest that degenerative spondylolisthesis does not significantly impact surgical outcomes.

### Spacers and Interspinous Devices 

Stand-alone interspinous spacers are designed for the treatment of symptoms of intermittent neurogenic claudication secondary to moderate lumbar spinal stenosis and.

are implanted by minimally invasive methods through a cannula. Interspinous spacers aim to provide indirect decompression of the lateral and central spinal canal without violating the spinal canal and to support the segments. Range of motion, flexion, and bending are preserved. There are currently only a few devices available on the US market.

One of the most well-known devices is called Superion^®^ Interspinous Spacer, also known as Vertiflex^®^, which is a titanium implant that can be placed between the spinous processes of the vertebrae to relieve pressure on nerves and blood vessels.

The outpatient procedure involves several steps. First, a small tube, or cannula, is inserted into the interspinous space. A sizing instrument then determines the appropriate implant size. Finally, the Vertiflex^®^ device is delivered through the cannula and deployed between the spinous processes of the affected lumbar level. Typically, the implant is placed at one or two adjacent levels between L1 and L5. The Vertiflex^®^ procedure is contraindicated in patients with grade > 1 spondylolisthesis, spinal fractures, scoliosis (Cobb angle > 10°), cauda equina syndrome, osteoporosis (T-score < -2.5), BMI > 40, or segmental ankylosis.

A study analyzing data from a Vertiflex^®^ registry [37] across 86 U.S. clinical sites demonstrated long-term improvements in both back and leg pain. The research showed that average leg pain severity decreased by 60%, from 76.6 ± 22.4 mm before the procedure to 30.4 ± 34.6 mm at the 12-month mark. The percentage of patients who responded positively to the treatment was 64% at 3 weeks, 72% at 6 months, and 75% at 12 months. Similarly, back pain severity improved by 48%, from 76.8 ± 22.2 mm preoperatively to 39.9 ± 32.3 mm at 12 months, with a 67% responder rate at the one-year follow-up.

More recently, concerns about the long term efficacy when compared to decompressive surgery, together with the increasing reports of challenging revisions and explantations, have contributed to reduced adoption in current practice [https://www.accessdata.fda.gov/scripts/cdrh/cfdocs/cfres/res.cfm? id=214586].

To note, newer interspinous and interlaminar fixation systems have emerged. For example, the ZIP™ minimally invasive interspinous fusion device (Aurora Spine) has received FDA clearance and is commercially available, reflecting the ongoing development in less-invasive posterior stabilization devices.

Another important minimally invasive device designed for interspinous- interlaminar fusion is the Minute Man G3 from Spinal Simplicity Inc. It aims to temporarily fix and stabilize the thoracic, lumbar, and sacral spine to promote eventual fusion and address dynamic stenosis. The device features bilateral locking plates that secure to both sides of the spinous process and contains bone graft material to facilitate fusion, providing immobilization and stability to the spinal segments.

For the placement, a small lateral to midline incision is made using fluoroscopic guidance. A series of instruments is then used to access the space between the vertebrae, partially remove the interspinous ligament, and dilate the space to the appropriate size. The MinuteMan G3^®^ device, packed with bone graft, is then inserted into the prepared interspinous and interlaminar space. Once in position, an extension plate is deployed, and the device is tightened against the spinous processes. Fluoroscopy confirms the correct placement before the incision is closed.

This procedure is indicated for Grade 1–2 spondylolisthesis, central and foraminal lumbar stenosis (not FDA-approved), and painful degenerative disc disease. There are currently no published prospective trials using the Minuteman® procedure.  

## Other devices

Lastly, another commonly used option is the Coflex^®^ device, which is a U-shaped titanium implant designed to treat moderate to severe lumbar spinal stenosis (LSS). It functions as a dynamic interlaminar implant, meaning it is placed between the lamina of adjacent vertebrae to provide stability after a decompression surgery while still allowing for natural movement at that level.

According to the manufacturer, the Coflex^®^ implant is intended for patients with moderate to severe LSS who might not achieve sufficient stability from decompression surgery alone but wish to avoid a more rigid spinal fusion procedure involving pedicle screws.

The implantation phase involves creating a 3 cm skin incision over the spinous process, preserving the supraspinous ligament and facet capsules while preparing the site with minimal bony resection. The implant is then inserted via impaction, confirmed with fluoroscopy, and the procedure is completed in under an hour, with patients mobilized shortly after and discharged within two days.

This procedure is indicated for neurogenic claudication or leg, buttock, or groin pain, with or without back pain relieved by flexion. Contraindications include fixed motor deficits, cauda equina syndrome, instability, prior lumbar surgery, significant neuropathy, scoliosis, fractures, severe osteoporosis, obesity, infection, systemic disease, or vertebral metastasis.

In the prospective, randomized, controlled trial [38] enrolling patients with moderate-to-severe lumbar spinal stenosis, with or without up to a grade 1 spondylolisthesis, which were randomized to receive Coflex^®^ vs. spinal fusion, at the three-year follow-up, a significantly larger percentage of patients in the Coflex^®^ Interlaminar Stabilization group had a clinically significant improvement of 15 points or more on the Oswestry Disability Index (ODI) compared to the spinal fusion group. Overall, composite clinical success at 36 months was achieved by 62.2% of Coflex^®^ patients versus 48.9% of fusion patients.

Table 2 contains a summary of the cited studies.

**Table 2:** Interspinous Spacer and Interlaminar Devices for Lumbar Spinal Stenosis

Device / Author, Year	Study Design	Follow-up	*N* Patients	Outcomes Assessment	Complications / Limitations	Notes
Superion^®^ / Vertiflex^®^ Nunley et al., [37]	Prospective registry study (86 US clinical sites)	3 weeks, 6 months, 12 months	Not specified (multi-site registry)	Leg pain decreased 60% (76.6→30.4 mm VAS at 12 mo); 75% responder rate at 12 mo. Back pain decreased 48% (76.8→39.9 mm VAS at 12 mo); 67% responder rate at 12 mo.	Not reported in detail. Subsequent concerns raised about long-term efficacy relative to decompressive surgery and challenges with revision/explantation leading to reduced adoption.	Titanium implant placed between spinous processes (L1–L5). CI: Grade > 1 spondylolisthesis, fractures, scoliosis > 10°, cauda equina syndrome, osteoporosis (T-score < − 2.5), BMI > 40, segmental ankylosis.
Coflex^®^ Interlaminar Stabilization Bae et al., [38]	Prospective, randomized, controlled trial (Coflex^®^ vs. spinal fusion)	36 months (3 years)	Not specified; moderate-to-severe LSS ± Grade 1 spondylolisthesis	Composite clinical success at 36 months: 62.2% (Coflex^®^) vs. 48.9% (fusion). Coflex^®^ group had significantly greater ≥ 15-point ODI improvement vs. fusion group.	Not explicitly detailed. Procedure involves 3 cm incision; patients mobilized shortly after and discharged within 2 days.	U-shaped titanium implant placed between laminae after decompression. CI: fixed motor deficit, cauda equina syndrome, instability, prior lumbar surgery, severe osteoporosis, obesity, infection, vertebral metastasis.
MinuteMan G3^®^ (Spinal Simplicity Inc.) No published prospective trial	No published prospective trials available	N/A	N/A	No outcomes data available from prospective trials.	N/A	Interspinous-interlaminar fusion device. Indicated for Grade 1–2 spondylolisthesis and central/foraminal stenosis (not FDA-approved for stenosis). Features bilateral locking plates with bone graft.
ZIP™ Interspinous Fusion Device (Aurora Spine) FDA Cleared; no published RCT	No published prospective RCTs; FDA cleared for commercial use	N/A	N/A	No outcomes data available from prospective trials.	N/A	Minimally invasive interspinous fusion device. Represents newer generation of less-invasive posterior stabilization. Long-term evidence pending.

### Endoscopic Decompression

Minimally invasive discectomy decompresses the spine via nucleotomy, indirectly reducing pressure on the exiting nerve root. Transforaminal endoscopic techniques, by contrast, can achieve decompression through either direct or indirect means, with the specific variant — interlaminar, transforaminal percutaneous endoscopic, or endoscopic lumbar foraminotomy — selected according to the pattern and location of stenosis [39]. For far-lateral and foraminal pathology, the transforaminal route tends to be favored, while the interlaminar approach better serves central and paracentral herniations, particularly at L5–S1 where a high-riding iliac crest can obstruct the transforaminal corridor.

Endoscopic decompression has been proven to be no less effective than other minimally invasive procedures for reducing leg pain in lumbar spinal stenosis. The three most commonly employed techniques are full endoscopy, microendoscopy, and biportal endoscopy, all of which rely on indirect visualization of the operative field through a camera introduced via a working channel.

### Transforaminal Endoscopic Lumbar Discectomy (TELD)

TELD relies on accessing the disc through the intervertebral foramen, threading instruments between the exiting and traversing nerve roots. The foramen itself is a dynamically changing space — bounded anteriorly by the disc, posteriorly by the facet joint, above and below by the respective pedicles, and medially by the thecal sac — whose dimensions shift with both spinal movement and progressive degeneration. Within this space run the spinal nerve and dorsal root ganglion, segmental vessels, lymphatics, sinuvertebral nerves, and surrounding fat, all of which must be negotiated during access.

Safe passage depends on working within Kambin’s triangle, a space whose anterior border is the exiting root, whose floor is the superior endplate of the vertebra below, and whose medial boundary is the thecal sac and traversing root as they are partially shielded by the facet. Cadaveric data from Mirkovic and colleagues established that a 6.3 mm working cannula can be safely accommodated at the mid-pedicular level, while a 7.5 mm cannula — the size of most commercially available systems — is tolerated at the medial pedicular line in anteroposterior fluoroscopic view.

The procedure is typically carried out under local anesthesia with intravenous conscious sedation, most commonly with the patient prone, which offers the most consistent anatomical orientation. Preoperative cross-sectional imaging guides both the skin entry point and the needle trajectory, which is planned to reach the herniated fragment by grazing rather than traversing the facet. Once the needle is positioned within the disc, a mixture of contrast dye and indigo carmine is injected: the dye outlines the disc architecture fluoroscopically and selectively stains the degenerated, acidic nuclear material blue, providing a useful intraoperative color marker during fragment retrieval. The needle is then exchanged over a guidewire for sequential dilators and a working cannula, through which the rigid endoscope is advanced.

Once inside, the surgeon orients by identifying the epidural fat, annular defect, posterior longitudinal ligament, and the traversing and exiting roots, as well as the superior facet and adjacent pedicle notches. Fragments are retrieved with pituitary-type forceps, and residual annular bleeding or tears are addressed with bipolar radiofrequency coagulation or holmium: YAG laser as needed. Adequate decompression is confirmed by restoration of free pulsatile movement of the thecal sac and nerve root, the appearance of fresh epidural bleeding, and resolution of the patient’s referred limb pain — a real-time feedback advantage afforded by performing the case under conscious sedation.

Two distinct philosophical approaches have shaped TELD technique over time. The “inside-out” method, refined by Yeung, begins with intradiscal work and progressively moves outward, systematically identifying pain generators within the foramen and disc, freeing both roots, performing fragmentectomy, and ablating annular tears. The “outside-in” approach popularized by Hoogland instead enters the epidural space directly after facet resection, requiring foraminoplasty to access the target fragment but carrying a higher risk of bleeding and reduced visualization during the approach phase.

Anatomically challenging variants require procedural modification. For far-lateral extraforaminal herniations, a steeper needle angle of up to 50°, a skin entry point 5–8 cm from the midline, and targeting directed at the superior endplate of the caudal vertebra are required. Migrated herniations demand corresponding shifts in the entry point — caudal for upward migration, cephalad for downward migration — while high-grade caudal migration may additionally require foraminoplasty or partial pediculotomy to establish adequate access to the sequestered fragment.

### Interlaminar Endoscopic Approach

At L5–S1, anatomical constraints frequently render the transforaminal route impractical — particularly in male patients with a high iliac crest, or when an upward-migrating herniation places the fragment out of line with any feasible transforaminal trajectory. The interlaminar window at this level is the widest in the lumbar spine, typically around 31 mm, and a modest overhang of the L5 lamina creates a natural working corridor. Preoperative imaging should characterize whether the herniation lies in a shoulder or axillary position relative to the nerve root, since this determines the angle of cannula placement: shoulder-type herniations require a more superomedially directed approach, while axillary herniations are better accessed by aiming toward the inferior lamina. After sequential dilation, the endoscope is advanced to the spinolaminar junction, the ligamentum flavum is carefully split to expose underlying epidural fat, and radiofrequency coagulation is used to clear the fat layer before the herniated fragment is identified and removed under direct vision, with the working cannula positioned to protect the traversing root throughout.

### Full Endoscopic, Microendoscopic, and Biportal Techniques

Full endoscopic systems consolidate the optic, irrigation, and instrument channels into a single 6–8 mm endoscope, enabling an entirely percutaneous workflow. Microendoscopic discectomy, developed in parallel by Destandau and Foley during the 1990s, uses sequential muscle-dilating tubes with an endoscope or operating microscope attached at the proximal end, preserving the posterior ligamentous tension band while providing a familiar instrument feel for surgeons transitioning from open surgery. The biportal technique separates the camera and working portals into two distinct skin incisions a few centimeters apart, which allows the surgeon to triangulate instruments freely and work with standard open surgical tools — a design choice that tends to lower the technical threshold compared to single-portal endoscopic systems. An additional advantage of the biportal approach is the capacity to perform bilateral decompression through a unilateral corridor using an “over-the-top” maneuver, avoiding a second incision while preserving the spinous process and interspinous ligaments.

A 2022 randomized controlled trial of 437 patients found no meaningful difference in clinical outcomes between these three decompression strategies at two-year follow-up [40], with Oswestry Disability Index and VAS scores for both leg and back pain improving equivalently across all groups. This suggests that technique selection is more appropriately driven by surgeon experience and the specific anatomy of the patient than by any intrinsic superiority of one approach.

In a recent retrospective single-institutional series [41], uniportal and biportal approaches produced comparably excellent outcomes for lumbar disc herniation, with similar operative durations, complication rates, and VAS reductions exceeding 3 points at twelve months in both cohorts.

### Cervical and Thoracic Applications

Once confined exclusively to the lumbar spine, endoscopic techniques now extend to cervical and thoracic pathology. Anterior percutaneous endoscopic cervical discectomy offers the distinct advantage of being performable under local anesthesia as an outpatient procedure, eliminating the need for segmental fusion and its downstream complications. The patient’s wakefulness throughout the case provides continuous neurological monitoring. Access is obtained through the natural interval between the carotid sheath laterally and the tracheoesophageal complex medially, with the needle introduced between the longus colli muscles to minimize sympathetic chain and vascular risk. Discography with contrast-indigo carmine mixture is again employed to delineate the target fragment, and after working cannula placement, fragments are extracted with forceps or accessed through an annular window created with a side-firing holmium laser for intracanalicular material.

Posterior endoscopic cervical foraminotomy — sometimes called keyhole foraminotomy — is an alternative suited to foraminal disc herniations and osteophytic stenosis where the anterior disc need not be disturbed. By removing the pathological fragment from behind, the approach sidesteps the need for discectomy and fusion entirely, and can also address cases where prior anterior cervical surgery has failed to resolve foraminal symptoms. Facet drilling is an inherent part of the decompression, but resection must remain below 50% of the joint to preserve segmental stability. The procedure is contraindicated in patients with axial neck pain, instability, or cervical kyphosis, all of which would be worsened by posterior element disruption.

Thoracic disc herniations represent a small fraction of disc disease overall — roughly 0.25–0.75% of all herniations — and even fewer require surgery. Percutaneous endoscopic thoracic discectomy approaches the disc from a posterior or posterolateral trajectory and avoids the chest wall incisions and postoperative drainage tubes inherent to thoracoscopic approaches. Its application is restricted to soft, non-calcified herniations, and it is contraindicated when ossification of the posterior longitudinal ligament, severe cord compression, or significant disc space collapse is present.

Table 3 contains a summary of the cited studies.

**Table 3:** Endoscopic Decompression Techniques for Lumbar (and Cervical/Thoracic) Spinal Stenosis

Technique / Author, Year	Study Design	Follow-up	*N* Patients	Outcomes Assessment	Complications	Notes
Full endoscopic vs. microendoscopic vs. biportal endoscopic decompression Hermansen et al., [40]	Prospective, multicenter randomized clinical trial	2 years	437	No meaningful difference in ODI or VAS (back and leg pain) among the three techniques at 2-year follow-up. Outcomes were equivalent across all groups.	Not separately detailed; technique selection driven by surgeon experience and patient anatomy rather than complication profile.	All three techniques rely on camera visualization via a working channel. Biportal approach allows bilateral decompression through unilateral corridor (“over-the-top” maneuver). JAMA Network Open 2022.
Uniportal vs. Biportal Endoscopic Interlaminar Approach (L5/S1 disc herniation) Zuo et al., [41]	Retrospective, single-institutional series	12 months	Not specified	Comparably excellent outcomes in both groups. VAS reductions exceeding 3 points at 12 months in both cohorts. Similar operative durations and complication rates.	Comparable complication rates between uniportal and biportal approaches; no significant difference reported.	Biportal approach uses two skin incisions (camera + working portal) a few centimeters apart, allowing free instrument triangulation and use of standard open tools. Frontiers in Surgery.
Transforaminal Endoscopic Lumbar Discectomy (TELD) (Technique review; Mirkovic et al. cadaveric data) [39, 42]	Technical description / cadaveric study	N/A	Cadaveric specimens	Established safe cannula sizing within Kambin’s triangle: 6.3 mm cannula at mid-pedicular level; 7.5 mm (commercially standard) tolerated at medial pedicular line. Adequate decompression confirmed by free pulsatile thecal sac movement and resolution of referred limb pain under conscious sedation.	Fluoroscopic dependence (radiation to patient and surgeon). Risk of nerve root injury if outside Kambin’s triangle. Steep learning curve; disorientation risk without tactile landmarks. [42]	Performed under local anesthesia + IV conscious sedation (prone). Indigo carmine dye selectively stains degenerated nucleus. Two philosophical approaches: Yeung “inside-out” vs. Hoogland “outside-in” (foraminoplasty required).
Percutaneous Endoscopic Cervical Discectomy (PECD) & Posterior Cervical Foraminotomy Tang et al., [42]	Narrative review / technique description	N/A	N/A (review)	PECD: outpatient, local anesthesia, avoids segmental fusion. Posterior cervical foraminotomy: addresses foraminal herniations and osteophytic stenosis without discectomy/fusion; can manage failed prior anterior surgery. Thoracic: avoids chest wall incisions of thoracoscopic approaches; limited to soft non-calcified herniations.	Posterior foraminotomy CI: axial neck pain, instability, cervical kyphosis. Facet resection must remain < 50% to preserve stability. Thoracic CI: OPLL, severe cord compression, disc space collapse. Patient wakefulness provides continuous neurological monitoring (PECD).	Thoracic disc herniations ~ 0.25–0.75% of all herniations. PECD access via interval between carotid sheath and tracheoesophageal complex. Endoscopic techniques now extend beyond lumbar spine. World J Orthop 2023.
Endoscopic Spine Surgery – Learning Curve & Complications Tang et al., [42]; Hermansen et al., [40]	Narrative review / pooled RCT analysis	Ongoing (procedure adoption)	N/A	No intrinsic superiority of one endoscopic technique over another demonstrated at 2 years [40]. Technique selection driven by surgeon experience and patient anatomy.	Working geometry inverted vs. open surgery (superficial-to-deep). Fluoroscopic dependence: cumulative radiation. Risk of insufficient decompression, inadvertent neural contact, wrong-level.	Recommended training pathway: standardized fellowship, cadaveric simulation, formal proctorship. Biportal approach lowers technical threshold vs. single-portal systems due to free instrument triangulation.

### Complications and Learning Curve

Beyond operative hazards, the learning curve for endoscopic spine surgery is a meaningful barrier to adoption. The working geometry is inverted relative to open posterior decompression — the endoscopic surgeon advances from superficial to deep, outside to in, rather than beginning at the canal and working outward. Fluoroscopic dependence adds cumulative radiation burden to both patient and surgeon, and the absence of tactile landmarks that are immediately familiar in open surgery can lead to disorientation, insufficient decompression, or inadvertent neural contact [42]. Standardized fellowship training, cadaveric simulation, and formal proctorship arrangements have been proposed as the most effective framework for compressing this curve and ensuring safe independent practice.

## Discussion

### The Evolving Treatment Landscape for Lumbar Spinal Stenosis

Lumbar spinal stenosis is the most common indication for spinal surgery in patients over 65, yet the optimal management strategy remains genuinely contested. The condition spans a wide clinical spectrum — from incidental radiological findings in asymptomatic individuals to debilitating neurogenic claudication that severely curtails walking tolerance and quality of life — and this heterogeneity makes uniform treatment recommendations difficult. No single therapy has emerged as categorically superior across all patient subgroups, and the decision between conservative and surgical management, and among the expanding portfolio of surgical options, continues to be driven largely by symptom severity, patient comorbidity, anatomical pattern of disease, and surgeon preference.

### Conservative Management

Non-surgical treatment remains the appropriate first-line strategy for the majority of patients with LSS, particularly those with mild to moderate symptoms, given the condition’s variable and often slowly progressive natural history. Structured physiotherapy, with an emphasis on lumbar flexion-based exercise and core stabilization, reduces the mechanical load on the posterior elements and tends to ease neurogenic claudication, though the effect is modest and often incomplete. Nonsteroidal anti-inflammatory drugs, membrane-stabilizing agents such as gabapentinoids, and oral corticosteroids are commonly prescribed, though the evidence supporting long-term pharmacological management is limited, and the side-effect burden in an elderly population is not trivial.

Epidural steroid injections occupy a contested but widely used position in the conservative algorithm. While they can provide meaningful short-term relief of radicular symptoms, their duration of benefit is typically weeks to a few months, and repeated injections carry diminishing returns alongside a risk of cumulative complications. Nonetheless, for patients who are poor surgical candidates or who wish to defer intervention, a course of epidural injections remains a reasonable bridging strategy.

Current evidence does not definitively establish that lumbar decompression surgery outperforms non-surgical treatment in reducing pain and disability associated with LSS. A 2020 Cochrane review [1] analyzed several clinical trials and found no significant benefit of surgery over non-surgical approaches — including physical therapy and medication — in reducing disability at six-month and one-year follow-ups, with the review itself judging the supporting evidence to be of low quality. A 2015 randomized trial of 169 participants [42] similarly found no superior outcomes in function or pain relief from surgery compared to physical therapy at two years. These findings have reinforced the recommendation of non-surgical approaches as the primary course of action, particularly in the early stages of disease [43, 44].

Conversely, more recent data have challenged this position. A 2021 single-blinded study of 63 patients [45] found that surgery produced greater improvements than non-surgical care at both two- and four-year follow-ups, suggesting that longer follow-up durations may reveal a surgical advantage that shorter trials obscure. This is consistent with the known natural history of LSS, in which conservative management can slow symptomatic progression but rarely reverses it, and in which prolonged neurogenic compression is associated with progressive motor and sensory deficit that may become increasingly difficult to reverse. For patients with moderate-to-severe symptoms, significant walking limitation, or evidence of neurological compromise, non-surgical management alone is unlikely to restore meaningful function, and timely surgical referral is warranted.

### Open Decompression: The Historical Standard

Open laminectomy or laminotomy with or without partial facetectomy and foraminotomy has been the standard surgical treatment for LSS for decades and remains the benchmark against which all newer techniques are measured. The procedure directly decompresses the neural elements by removing the lamina, hypertrophied ligamentum flavum, and osteophytic overgrowth under direct magnified visualization, with established efficacy and a well-characterized complication profile. Large registry and cohort data consistently demonstrate meaningful improvements in leg pain, walking capacity, and quality of life, with durable benefit extending to five and ten years in carefully selected patients.

The limitations of open decompression are primarily those of access. Midline muscle incision, epidural bleeding, and disruption of posterior bony and ligamentous stabilizers contribute to significant postoperative pain, prolonged recovery, and a risk of iatrogenic instability — particularly when bilateral facetectomy exceeds 50% of the joint. In the elderly and medically frail population that most commonly presents with LSS, these access-related morbidities are not trivial. Estimated blood loss, length of stay, and the anesthetic demands of open surgery must be weighed against the completeness of decompression it affords.

### The Role of Fusion

The question of whether to add fusion to decompression has been among the most debated in spine surgery. For patients with concomitant degenerative spondylolisthesis, early evidence from the SPORT trial [46] and subsequent analyses suggested that fusion alongside decompression produced superior outcomes compared to decompression alone, leading to widespread adoption of combined procedures in this population. However, the landmark SLIP trial [47] and the Swedish Spinal Stenosis Study [48] subsequently challenged this, finding that decompression alone produced equivalent outcomes to decompression with fusion at two and five years in patients with stable low-grade spondylolisthesis, without the additional operative time, blood loss, and implant-related complications associated with instrumented fusion.

For pure stenosis without instability or spondylolisthesis, the evidence does not support routine fusion. Adding instrumentation increases procedural morbidity, cost, and the risk of adjacent segment disease without a corresponding clinical benefit in this group. Fusion remains most clearly indicated when there is dynamic instability on flexion-extension imaging, significant sagittal imbalance requiring correction, or when decompression itself necessitates removal of stabilizing structures to an extent that would predictably generate postoperative instability.

### Minimally Invasive Surgical Decompression

The development of minimally invasive surgical (MIS) techniques has substantially altered the risk-benefit calculus of surgical intervention in LSS, particularly for older and higher-risk patients. By replacing midline muscle stripping with paramedian muscle-splitting or tube-based dilation approaches, MIS decompression achieves canal and foraminal decompression comparable to open surgery while substantially reducing blood loss, postoperative pain, inpatient stay, and recovery time. Meta-analyses and several randomized trials have confirmed that tubular microdiscectomy and minimally invasive laminotomy produce equivalent clinical outcomes to open procedures with a more favorable perioperative profile, though the effect on long-term reoperation rates remains an active area of investigation.

A 2022 randomized trial of 437 patients [40] found no significant difference in outcomes among these three modalities at two years, and the choice between them is therefore largely a function of training, institutional familiarity, and case complexity. What unites them is the principle of targeted decompression: removing precisely the tissue responsible for neural compression without the collateral disruption of stabilizing structures that open laminectomy entails.

MIS techniques are not without drawbacks. The learning curve is steep, the operative field is constrained, and fluoroscopic guidance requirements increase radiation exposure to both patient and surgeon. The limited visualization afforded by narrow working channels can make it technically demanding to confirm adequacy of decompression, and the absence of familiar anatomical landmarks increases the risk of wrong-level surgery and missed pathology. These limitations are most pronounced early in a surgeon’s experience and generally diminish with volume, but they underscore the importance of structured training pathways and proctorship for surgeons adopting these techniques.

### Percutaneous Indirect Decompression (PILD) Procedure

The PILD procedure occupies a distinct and somewhat narrower niche within this treatment landscape. Rather than directly addressing neural compression, it reduces the contribution of ligamentum flavum hypertrophy to central canal stenosis by percutaneously debulking the thickened ligament via a posterior interlaminar approach, using a series of specialized cutting and aspiration instruments under fluoroscopic guidance. The procedure carries a minimal tissue trauma profile, requires no general anesthesia, and can be performed as an outpatient intervention, making it an attractive option for patients whose comorbidity burden makes conventional surgery prohibitive.

Its clinical value, however, is defined by the indication. PILD is appropriate only when central canal stenosis is predominantly attributable to ligamentum flavum hypertrophy, a subset that must be carefully identified on preoperative MRI by direct measurement of ligament thickness — typically exceeding 2.5 mm per side in the trial populations that established its efficacy. Several studies have demonstrated that ligamentum flavum thickness varies considerably across both normal and stenotic spines, with documented values up to 4 mm per side, and this variability can influence both patient selection and the number of levels treated [49].

The procedure is not indicated for stenosis driven by disc prolapse, facet joint hypertrophy, foraminal narrowing, or spondylolisthesis, nor can it be offered to patients who have previously undergone surgery at the target segment. These constraints substantially limit the proportion of the LSS population for whom PILD is a viable primary intervention. Patients with grade 1 or higher spondylolisthesis, dynamic instability, or multilevel disease with a significant non-ligamentous component are not candidates, and in these groups, either MIS decompression or open surgery with or without fusion will remain necessary.

A 2025 retrospective study by Chu et al. [50] compared 175 patients treated with PILD against a propensity-matched cohort of 175 patients undergoing open lumbar decompression and found that those patients had nearly twice the likelihood of requiring reoperation and a higher incidence of postoperative neurological disturbances, including neuritis and sensory changes. The study’s retrospective design, absence of direct stenosis severity matching, and failure to account for etiology — particularly the presence or absence of spondylolisthesis — limits the strength of its conclusions, but it reinforces the need for prospective head-to-head trials that rigorously control for disease characteristics. Reoperation rates following PILD have been reported at up to 7% within the first year of the procedure [31, 51], a figure that is clinically significant given that one of the implicit goals of a minimally invasive percutaneous approach is precisely to delay or avoid more invasive subsequent surgery.

To date, no study has directly compared PILD with MIS endoscopic or open laminectomy approaches in a prospective randomized design, a gap that reflects both the differing patient populations that each technique serves and the challenges of performing equipoise-based trials across procedures with substantially different eligibility criteria. This limits the ability to position mild^®^ definitively within the surgical hierarchy and underlines the importance of ongoing independent evaluation of its clinical efficacy and cost-effectiveness.

### Toward a Patient-Centered Treatment Algorithm

Taken together, the available evidence supports a stepwise and individualized approach to LSS management. Conservative treatment — structured physiotherapy, pharmacological management, and selective use of epidural steroid injections — is the appropriate starting point for most patients and should be pursued for a minimum of 8–12 weeks before surgical referral is considered in the absence of progressive neurological deficit. For patients who fail conservative management, the choice of intervention should be guided by the anatomical pattern of stenosis, the presence or absence of instability, comorbidity profile, and patient goals.

Patients with isolated ligamentum flavum hypertrophy, no instability, and significant medical comorbidities may be well served by mild^®^ or other PILD strategies. Those with more complex or multilevel stenosis, facet-driven or discogenic compression, or mild spondylolisthesis without instability are better candidates for MIS or open direct decompression, with fusion reserved for cases where instability is documented or decompression itself would create it. Open laminectomy remains an important option when the extent of decompression required exceeds what can be reliably achieved through a tubular or endoscopic corridor, and for surgeons without extensive MIS training. As surgical techniques continue to evolve and longer-term comparative data mature, refining these indications will depend heavily on well-designed prospective trials that stratify patients by the precise disease pathophysiology rather than pooling heterogeneous LSS populations under a single surgical label.

## Conclusion

The PILD procedure can significantly reduce pain intensity and improve functional status. It is a safe procedure with no major procedure-related complications and has the advantage of minimal invasiveness and non-interference with future surgery. It is accepted as an option for LSS patients who failed conservative treatment; however, its long-term efficacy is still uncertain. Additionally, PILD, and mild^®^ in particular, have not been directly compared to all currently available minimally invasive treatments such as endoscopic decompression, thus its place in the continuum of available treatment options requires ongoing investigation.

##  Key references

- “Deer, T. R., Chafin, T. B., Costandi, S. J., Qu, H., Kim, C., Jassal, N., ... & Calodney, A. (2024). The MOTION study: Two‐year results of a real‐world randomized controlled trial of the mild® procedure for treatment of lumbar spinal stenosis. Pain Practice, 24(1), 109-119. “
  - ○ This study is relevant because it provides high quality, two year randomized controlled trial data demonstrating the sustained effectiveness and safety of the mild® procedure for lumbar spinal stenosis in a real world population. Its long term outcomes strengthen the evidence base for mild as a minimally invasive, durable alternative for patients who are not ideal candidates for more extensive surgical interventions.
- “Chu, T., Johnson, S. E., Willis, K., Nathani, K. R., Pennington, Z., Graepel, S. P., ... & Bydon, M. (2025). Minimally invasive lumbar decompression versus open decompression for lumbar spinal stenosis: a propensity score–matched analysis. Journal of Neurosurgery: Spine, 1(aop), 1-7. “
  - ○ This study is relevant because it is a large real world, non–industry sponsored investigation comparing minimally invasive lumbar decompression with traditional open surgery using a rigorous propensity score matching. Although it is a retrospective study, its independent design offers a valid assessment of how these procedures perform in everyday clinical practice.

## Data Availability

No datasets were generated or analysed during the current study.
